# How sweet is your love? Disentangling the role of marital status and quality on average glycemic levels among adults 50 years and older in the English Longitudinal Study of Ageing

**DOI:** 10.1136/bmjdrc-2022-003080

**Published:** 2023-01-03

**Authors:** Katherine J Ford, Annie Robitaille

**Affiliations:** 1 Institute for Research on Socio-Economic Inequality (IRSEI), University of Luxembourg, Esch-sur-Alzette, Luxembourg; 2 Interdisciplinary School of Health Sciences, University of Ottawa, Ottawa, Ontario, Canada; 3 Perley Health Centre of Excellence, Ottawa, Ontario, Canada

**Keywords:** Social Support, Longitudinal Studies, Diabetes Mellitus, Type 2, Primary Prevention

## Abstract

**Introduction:**

The health benefits of marriage have been widely documented and, to a lesser extent, the effects of marital quality. Marital relationships may be particularly relevant to the health of older adults. This study explores the associations of marital status and marital quality with average glycemic levels in older adults using longitudinal data.

**Research design and methods:**

Our sample consisted of adults aged 50–89 years without previously diagnosed diabetes from the English Longitudinal Study of Ageing (n=3335). We used biomarker data from waves 2 (2004/2005), 4 (2008/2009) and 6 (2012/2013) to analyze changes in hemoglobin A1c (HbA1c) levels within individuals in relation to their marital indicators (marital status, social support from spouse, and social strain from spouse) over time using linear fixed effect models.

**Results:**

We found that being married was associated with lower HbA1c values (β: −0.21%; 95% CI −0.31% to −0.10%) among adults without pre-existing diabetes. Spousal support and spousal strain were generally not associated with HbA1c values.

**Conclusions:**

It seems that marital relationships, regardless of the quality of the relationship, are associated with lower HbA1c values for male and female adults aged over 50 years.

**WHAT IS ALREADY KNOWN ON THIS TOPIC**

- Social health indicators, such as social network size, have varied associations with type 2 diabetes incidence and prevalence.

**WHAT THIS STUDY ADDS**

- Spouses/cohabitating partners may be a particularly important type of relationship and source of social support and/or strain for adults in mid to later life, thus we explore the specific benefits of this type of relationship among older adults.
- We found that having a spouse/partner was associated with lower average glycemic levels in those without pre-existing diabetes, while indicators of marital quality (spousal support/strain) generally did not appear to have significant associations with hemoglobin A1c (HbA1c) levels.

**HOW THIS STUDY MIGHT AFFECT RESEARCH, PRACTICE OR POLICY**

- Practitioners could consider that older adults without pre-existing diabetes who are experiencing marital/cohabitating partnership transitions may be at particular risk of worsening glycemic levels.

## Introduction

Social health is a multidimensional construct of significant relevance to older adults.^1^ Type 2 diabetes risk has been associated with a number of social health dimensions including social isolation, loneliness, living arrangements, social support, and social network size.^2–9^ However, the effects of each specific social health dimension are varied and complex. For example, some have found an effect of social network size on type 2 diabetes risk,^2^ while others have not.^4 10^ Furthermore, the types of relationships in one’s social network seem to matter, with marital relationships having seemingly protective effects, unlike religious, club, or other family ties.^4^ Moreover, living with someone also appears to be protective, but possibly more so for men.^2 5 7^

Evidence suggests that social relationships evolve over the life course with regular contact with children and friends waning into mid-life,^11^ potentially increasing the salience of a ‘life’ partner. A meta-analysis of social support and mortality risk further purports that support from family is more beneficial than support from friends,^12^ while others suggest that friends may be more influential on health behaviors in adolescence and partners more so in adulthood.^13^ One could assume that spouses and cohabitating partners would also spend more time with each other than with other types of relations given shared living spaces, further implying a larger dose with relationship indicators.

Marital relationships have been extensively associated with positive health effects.^14–18^ One study found that marriage had protective effects on health in mid-life, even after accounting for selection effects into marriage.^14^ Others have found that the negative health effects of social isolation were mainly driven by marital/cohabitating status, specifically with regard to inflammatory markers and blood pressure.^16^ However, one could expect that the quality of a marriage also matters for health outcomes. Another study suggested that happy marriages had protective effects on blood pressure profiles, yet unhappy marriages were not an advantage over being single.^15^ Moreover, social support from friends among single people did not mitigate differences with those who were happily married.^15^ Further research found that marital strain was particularly detrimental to self-reported health for older adults and demonstrated that the relationship was primarily in the direction of marital quality influencing health rather than the other way around.^17^

Marital relationships could be causally linked to type 2 diabetes risk and worsening glycemic regulation through stress buffering mechanisms, social regulation processes, and through socioeconomic pathways.^18–20^ The stress buffering effect of a supportive companion may both reduce the perceived gravity of a stressor and improve one’s perceived ability to cope with a given stressor.^20^ Thus, the inflammatory processes associated with stress may be reduced, with the physiological stress response having been directly linked to type 2 diabetes risk.^21 22^ On the other hand, social strain may increase stress and/or negatively impact the perceptions of its severity or one’s ability to cope with its demands. Social regulatory or social ‘contagion’ mechanisms work by influencing an individual’s health behaviors through norms within a social unit.^19 20^ Marital relationships in particular also act as small insurance policies and economies of scale, where partners share resources and assets.^18^ The income and wealth benefits of marriage could also be expected to support better health outcomes through the purchase of goods and services that promote health and reduced financial insecurity.

Many studies exploring type 2 diabetes risk use self-reported diagnoses as their outcome variable as this information is easy to collect in large population-based surveys with self-reported data. The drawback to using diagnoses is that timely diagnoses of type 2 diabetes are associated with healthcare usage,^23^ and healthcare usage is socially patterned.^24^ There may be significant underdiagnoses in sections of the population that are not regularly visiting their doctor. Furthermore, the International Diabetes Federation estimates that 1 in 3 people with diabetes are undiagnosed in Europe, and that ratio climbs close to 1 in 2 worldwide.^25^ Biomarkers, such as hemoglobin A1c (HbA1c), may provide a more accurate picture of an individual’s true metabolic state than self-reported diagnoses when collected as part of population-based surveys. HbA1c reflects average blood glucose levels over the past 2–3 months and does not require the individual to fast beforehand, unlike fasting blood glucose tests that require a fasted state and reflect blood glucose levels in the moment.^26^ Elevated HbA1c values are also associated with increased risk of hypoxia, cardiovascular disease, and mortality in non-diabetic populations,^26 27^ demonstrating its relevance to health beyond type 2 diabetes.

This study aims to explore associations between marital status or marital quality and HbA1c outcomes, rather than self-reported diagnoses of diabetes. In a previous study by Maki,^6^ relationship quality and HbA1c outcomes were analyzed using cross-sectional data from a sample of married American adults aged 33–83 years. It was found that spousal strain was not related to HbA1c values, while spousal support improved individuals’ perceived control over their health with associated improvements in HbA1c values.^6^ We build on this work by using longitudinal biomarker data from adults aged 50 years and older in the population-based English Longitudinal Study of Ageing reflecting an older adult population and their associated glycemic changes over time. Given evidence that suggests type 2 diabetes patient profiles differ somewhat between those with earlier versus later diagnoses^28 29^ and that marital status and quality may have amplified effects in older adults,^14 17^ our study will focus on the proposed marital health advantage for older adults that may be less evident in younger populations. We first hypothesize that the presence of a spouse/cohabitating partner will reduce HbA1c values, reflecting better glycemic regulation of married/cohabitating individuals. We further hypothesize that increases in spousal/partner strain will be associated with increased HbA1c values reflecting a shift toward greater glycemic disequilibrium for those who are married/cohabitating. Conversely, we hypothesize that spousal/partner support will be protective against HbA1c increases.

## Research design and methods

### Sample

This study used data from the English Longitudinal Study of Ageing (ELSA) (dataset).^30^ ELSA is a population-based sample of adults aged 50 years and older and their partners, who live in England. Data are collected biennially, with biomarker data collected in every other wave.^31^ Our sample consisted of respondents without pre-existing diabetes between the ages of 50 and 89 years in wave 2 (2004/2005)—when biomarker data were first available in ELSA. Pre-existing diabetes was determined by self-reports. This definition was chosen as we were concerned about medical or behavioral lifestyle changes associated with a known diagnosis.

**Figure 1:**
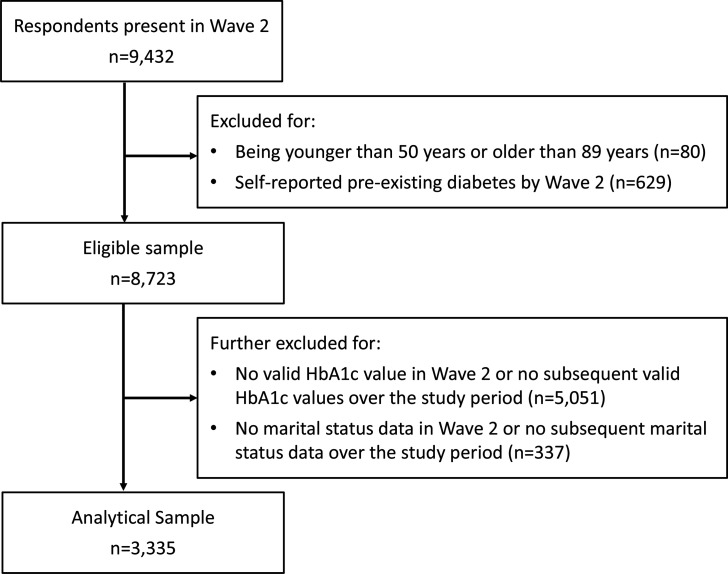
Sample selection flowchart. HbA1c, hemoglobin A1c.

All core respondents of ELSA were invited to have a nurse visit following the main interview in waves 2, 4 (2008/2009) and 6 (2012/2013). Blood samples were drawn at these visits. Partners of core respondents only had a nurse visit if they specifically requested one. In wave 2, no partners had a nurse visit and 87% of the core sample agreed to one.^32^ Eligible respondents were further excluded for having no follow-up biomarker data in waves 4 and 6, and for having no marital status information in wave 2 or no follow-up data on marital status. Figure 1 provides details of our sample selection. Those excluded were more likely to be older, physically inactive, current smokers, and in lower income quintiles. They were also more likely to have depression, higher BMIs, and no current employment (all p values <0.01). Those excluded were no different in terms of gender (p=0.10).

### Exposures

Respondents were first asked if they had a husband, wife, or partner with whom they live (yes/no) to indicate marital/cohabitating status, hereafter referred to as marital status. Social strain and social support within the marital/cohabitating relationship were measured by averaging the responses to three questions with a 4-point scale for those reporting having a spouse/partner. Social support, hereafter referred to as spousal support, was reflected in the following questions: *How much respondent feels their spouse/partner understands their feelings*, *how much respondent can rely on spouse/partner if they have a serious problem*, and *how much respondent can open up to their spouse/partner if they need to talk*. Social strain, hereafter referred to as spousal strain, was reflected in the following questions: *How much the spouse/partner lets the respondent down*, *how much the spouse/partner criticizes the respondent*, and *how much the spouse/partner gets on the respondents’ nerves*. If the respondent only answered two of the three questions on spousal support or strain, we took the average of two questions to retain more of the sample. Spousal support and strain were treated as continuous variables.

### Outcome

HbA1c was collected during the nurse visit following written informed consent for a blood sample to be drawn. Blood samples were analyzed at external laboratories. HbA1c values can be expressed in mmol/mol units or in percentage unit.^26^ Wave 6 HbA1c values were provided in mmol/mol unit instead of as a percentage like in the other waves, we thus converted the values to percentage units for this wave using HbA1c %=(HbA1c mmol/mol+23.5)/10.93.

### Confounders

Time-varying confounders included age, income, having employment, currently smoking, being physically active, having depression, body mass index (BMI), and having other social relationship types in the respondent’s social network (child, other immediate family, friend). Having employment, currently smoking, being physically active, having depression and other social relationship types were binary variables, while age, income, and BMI were treated as continuous variables. Being physically active was defined as moderate or vigorous activity at least once per week. Having depression was defined as six or more affirmative responses to the eight-item Centre for Epidemiological Studies Depression Scale.^3^ Income was measured in quintiles of equivalized household income. We used binary indicators for the presence of other types of relationships in the respondents’ social networks as other relationship types may substitute for the absence of a spouse/partner and spousal support, or may buffer the negative effects of spousal strain, thus confounding the effects of marital status. Respondents were asked if they had any children, other immediate family members, or friends.

### Analytical plan

Means with t-tests and proportions with Pearson χ^2^ tests were used to describe our sample. We used linear fixed effect models with our panel data to account for unmeasured time-invariant confounding using a mean-centering approach.^33^ We first modeled the crude relationship between marital status on HbA1c, adjusting only for age. We further adjusted the model for other time-varying socioeconomic and health confounders (model 2). Finally, we adjusted for the presence of other social relationships in model 3. In a fourth model, we added weights. Weights were constructed by taking the inverse probability of having at least one follow-up occasion in wave 4 or 6, multiplied by the wave 2 cross-sectional weight for having a blood sample drawn. We used known factors associated with attrition in ELSA to model the probability of remaining in the sample with a logistic regression: age, education, occupational class, wealth, and having a limiting health condition.^31^

For spousal support and strain, we excluded all observations where there was no reported spouse or partner living with the respondent. The same four models were used to analyze spousal support and strain. In models 3 and 4, the presence of specific types of social relationships (child, other immediate family members, friends) were replaced with perceived support or strain from those relationships, thus accounting for other sources of social support or strain than one’s spouse/partner. All statistical procedures were carried out using Stata V.13.1 (College Station, Texas, USA). Please see online supplemental appendix 1 for details on the codes used in the main analysis.

10.1136/bmjdrc-2022-003080.supp1 Supplementary data

### Supplementary analyses

We first tested gender interactions given previous studies that find effects of living alone on type 2 diabetes risk in men but not women.^2 7^ We also ran analyses excluding those who go on to be diagnosed with diabetes (self-reported) in waves 4 and 6, as treatment regimens may change HbA1c values through alternate or modified pathways. We present the main results including those who go on to be diagnosed with diabetes to avoid conditioning on the outcome. Finally, we computed odds ratios (ORs) for pre-diabetes and diabetes with fixed effect logit models for all significant associations between HbA1c levels and marital status or quality indicators. The threshold for pre-diabetes was defined as having HbA1c values at or above 5.7%, while diabetes was defined as having HbA1c values at or above 6.5% in line with the American Diabetes Association guidelines.^34^ We present the linear fixed effect models in the main analysis due to limitations with the fixed effect logit models, such as the inability to use probability weights with these models in Stata and smaller sample sizes with the less variable binary outcomes. Fixed effect models compute estimates over individuals who vary in their outcome over the study period, thus the binary coding of (pre-)diabetes limits the number of individuals who change in their outcome in comparison with fine grained changes in continuous HbA1c values.^35^

## Results

In wave 2 (2004/2005), 76% of the sample were married/cohabitating. Table 1 describes the sample at baseline according to their marital status. Those who were unpartnered at baseline were more likely to be older, female, in lower income quintiles, have depression, and currently smoke; and less likely to be physically active, currently employed, have children or other immediate family members (all p values <0.05).

**Table 1:** Descriptive details of the study sample from the English Longitudinal Study of Ageing at baseline in 2004/2005 (n=3335)

	Married/cohabitating (n=2524) % (n)/mean (SD)	Unpartnered (n=811) % (n)/mean (SD)	P value*	Missing N
Male	49% (1245)	28% (226)	0.000	0
Income quintile				
1	10% (243)	27% (219)	0.000	45
2	15% (368)	20% (162)		
3	20% (486)	20% (161)		
4	26% (635)	18% (148)		
5	30% (748)	15% (120)		
Currently working	45% (1147)	26% (211)	0.000	0
Depression	4% (89)	10% (81)	0.000	16
Physically active	84% (1646)	79% (547)	0.001	688
Currently smoking	12% (295)	16% (130)	0.001	6
Friends	96% (2419)	96% (778)	0.952	20
Other immediate family	95% (2378)	92% (745)	0.015	19
Children	92% (2323)	77% (622)	0.000	9
Spousal support (out of 4)	3.6 (0.5)	n/a	n/a	15
Spousal strain (out of 4)	1.8 (0.6)	n/a	n/a	16
BMI	27.7 (4.5)	27.4 (5.1)	0.208	93
Age	63.0 (7.5)	67.5 (9.3)	0.000	0
HbA1c in %	5.47 (0.53)	5.52 (0.49)	0.006	0
HbA1c value at/above pre-diabetes threshold (≥5.7%)	616 (24%)	231 (28%)	0.020	0
HbA1c value at/above pre-diabetes threshold (≥6.5%)	60 (2%)	28 (3%)	0.096	0

*t-tests for continuous variables and Pearson χ^2^ tests for categorical variables without the missing category.

BMI, body mass index; HbA1c, hemoglobin A1c.

For those that were married/cohabitating at baseline, the probability of transitioning to being unmarried/unpartnered was 5.1%, while the probability of transitioning to a married/partnered state for those that were not married/cohabitating at baseline was 3.5% (data not shown in the tables). Within-person variation accounted for 9.9% of the overall variability in marital status over the sample. This percentage was 20.4% for spousal support and 25.0% for spousal strain (data not shown in the tables). Table 2 displays the effect of a change in marital status, spousal support, and spousal strain on HbA1c values. Marital/cohabitating relationships were associated with a lowering of HbA1c values by 0.21% (95% CI −0.31% to −0.10%), indicating better glycemic regulation. Spousal support and spousal strain did not appear to have significant associations with HbA1c values, except with the base model for spousal strain that suggested an increase in HbA1c values by 0.04% (95% CI 0.01% to 0.07%) with increasing strain.

**Table 2:** Marital status, spousal support, spousal strain, and its associated effects on hemoglobin A1c values (%) in English adults aged 50–89 years

	Model 1* β (95% CI)	Model 2† β (95% CI)	Model 3‡ β (95% CI)	Model 4§ β (95% CI)
Marital status	−0.18 (−0.24 to −0.13)	−0.20 (−0.28 to −0.12)	−0.18 (−0.26 to −0.10)	−0.21 (−0.31 to −0.10)
Spousal support	0.01 (−0.03 to 0.04)	0.01 (−0.05 to 0.04)	0.00 (−0.05 to 0.06)	0.00 (−0.05 to 0.06)
Spousal strain	0.04 (0.01 to 0.07)	0.02 (−0.02 to 0.05)	0.04 (−0.01 to 0.08)	0.04 (−0.01 to 0.08)

*Model 1 was adjusted for age.

†Model 2 was adjusted for age, income quintile, current work, BMI, depression, smoking, physical activity.

‡Model 3 was further adjusted for the presence of family, friends, and/or children with marital status as the exposure. With spousal support or strain as the exposure, further adjustments were made for support or strain from family, friends, and/or children.

§Model 4 included weights.

BMI, body mass index.

With the supplementary analyses, we found no significant interactions between marital status or dimensions of marital quality and gender at the level of p<0.05. Furthermore, when we excluded those who went on to be diagnosed with diabetes in subsequent waves, we did not find any notable deviations from the main results. For analyses with the binary outcomes, 44% of those who initially did not have pre-diabetes transitioned to having HbA1c values above the pre-diabetes threshold by the end of the study period, while 7.5% of those above the threshold at baseline had values below the threshold at the end. For the diabetes cut-off, only 3.5% transitioned to HbA1c values above the cut-off, while 18% of those initially in the diabetes range had values below the cut-off by the end of the study period. We found significantly reduced odds of pre-diabetes among those in marital/cohabitating relationships across the three unweighted models, although the ORs for diabetes were not significant (table 3).

**Table 3:** Marital status and its associated ORs for (pre-)diabetes among English adults aged 50–89 years

	Model 1* OR (95% CI)	Model 2† OR (95% CI)	Model 3‡§ OR (95% CI)
Pre-diabetes	n=1452 0.49 (0.28 to 0.84)	n=1216 0.41 (0.19 to 0.88)	n=1185 0.43 (0.20 to 0.93)
Diabetes	n=200 0.57 (0.17 to 1.90)	n=154 0.70 (0.14 to 3.61)	n=150 0.68 (0.13 to 3.55)

*Model 1 was adjusted for age.

†Model 2 was adjusted for age, income quintile, current work, BMI, depression, smoking, physical activity.

‡Model 3 was further adjusted for the presence of family, friends, and/or children.

§Probability weights are not possible with ‘xtlogit’ in Stata.

BMI, body mass index.

## Discussion

We found that marital status, unlike marital support or strain, seemed to influence average glycemic levels in our sample of English adults aged 50 years and older without pre-existing, self-reported diabetes. Marital/cohabitating relationships were associated with a 0.21% decrease in HbA1c levels in this group. To contextualize our result, other work has suggested that a decrease of 0.2% in the population average HbA1c value would decrease excess mortality by 25%.^27^ Identifying and addressing barriers that impede the formation of romantic partnerships for older adults that wish to pursue these types of relationships may have subsequent benefits for glycemic levels in this population at risk for type 2 diabetes.^36^ Ageism, stereotypes of ‘asexual’ older adults, the deterioration of physical and mental health, and a lack of social opportunities are all cited barriers to dating and social connectedness among older adults.^37 38^

The null effect of perceived spousal support on glycemic outcomes was counter to our hypothesis, yet it could be that perceived spousal support is less relevant in routine day-to-day interactions than concrete support when people become aware of pre-diabetes or diabetes diagnoses. In other words, with routine circumstances, perceived support is not critical to glycemic regulation in individuals without diabetes, while when a diagnosis arrives tangible support may in fact be quite consequential. This squares with a review which suggested quality relationships were of importance for diabetes management in subjects with diagnoses.^39^

Our results differ somewhat with the study by Maki,^6^ where it was found that social support from friends or family had inverse associations with HbA1c, while strain was not related to HbA1c values. Their sample was younger and cross-sectional, which may explain differences in results. It is plausible that social support exerts its effects more prominently earlier in life course, with lower initial HbA1c levels for those who had greater social support earlier on. Our study can only provide a picture reflecting the effects of marital/cohabitating relationships on average glycemic levels for adults between the ages of 50 and 89 years. On the one hand, we can account for time-invariant confounders—measured or not—with the fixed-effects models employed in this study. Fixed-effects models compute effect sizes from within-person variation over time, while non-varying parameters are eliminated.^33^ Time-invariant confounders which may be particularly relevant to this line of research could include a family history of type 2 diabetes or stable personality factors that may influence perceptions of support and strain.^20 36^ Our modeling strategy was possible given the longitudinal data available in ELSA, but would not have been feasible with the cross-sectional data used in the study by Maki.^6^

Analyses with gender interactions reiterated the benefit of marriage/cohabitating to average glycemic levels for both men and women. Further analyses suggested that marital/cohabitating relationships were also protective against pre-diabetes, a glycemic range where individuals are considered to be at an increased risk of future diabetes and cardiovascular disease.^34^ However, we did not see an association with marital/cohabitating relationships and increased odds of diabetes. Others have found an increased risk of type 2 diabetes over a 22 year follow-up period among unmarried male health professionals aged 40–75 years who were living in the USA.^40^ This sample of highly educated men in the health field is not likely representative of most older adults, although the large sample size, long follow-up time, and different modeling strategy may have been better powered to detect effects.

### Strengths and limitations

One strength of our study was the use of HbA1c as an outcome measure versus self-reported diagnoses given the social patterning to healthcare usage, which is needed for diagnosing medical conditions. HbA1c was regularly collected as part of the survey protocol in ELSA, providing us with data for a longitudinal analysis of this indicator of diabetes status. Furthermore, the fixed effects models allowed us to account for time-invariant confounders that have the potential to bias estimates.

One limitation of this study was the sizeable attrition of subjects over the waves of ELSA with biomarker data. More than half of the wave 2 sample had no follow-up data and thus were excluded, increasing the potential for attrition bias. We included weighted analyses to address this limitation with our linear fixed effect models. Yet, probability weights with fixed effect logit models were not possible, and attrition bias may have been particularly limiting for detecting potential relationships between marital/cohabitating relationships and diabetes as a binary outcome.

Although our analytical strategy does not allow for causal claims, our assumptions and rationale for this study were that dimensions of the marital relationship influence HbA1c. The reverse, however, is another potential explanation that cannot be entirely ruled out. Higher HbA1c levels could elicit some degree of diabetes symptomology—including fatigue, thirst, blurred vision, and slow healing wounds^36^—which may in turn affect marital status or spousal strain if an ongoing deterioration in health increases irritability. Indeed, there is some evidence for health selection effects into marriage dissolution, whereby those in worse health are more likely to get divorced.^14 41^ However, this mechanism seems an unlikely explanation for our results given that symptoms of type 2 diabetes can be mild or absent for many years.^34 36^ We also ran a sensitivity analysis excluding all participants who went on to develop diabetes over the study period and found no significant deviations from our main results.

## Conclusions

This study focused on the association of marital/cohabitating relationships with average glycemic level in older adults, following evidence that suggests the types of relationships within one’s social network and the sources of social support and social strain matter for health outcomes.^12^ By focusing on this specific relationship type, we teased out its relevance to average glycemic levels from general social support, strain, and network size. Overall, our results suggested that marital/cohabitating relationships were inversely related to HbA1c levels regardless of dimensions of spousal support or strain. Likewise, these relationships appeared to have a protective effect against HbA1c levels above the pre-diabetes threshold. Increased support for older adults who are experiencing the loss of a marital/cohabitating relationship through divorce or bereavement, as well as the dismantling of negative stereotypes around romantic relationships in later life, may be starting points for addressing health risks, more specifically deteriorating glycemic regulation, associated with marital transitions in older adults.

## Data Availability

Data are available in a public, open access repository. ELSA data are available from the UK Data Service: https://doi.org/10.5255/UKDA-SN-5050-24.
