# Supplementary material for: How sweet is your love? Disentangling the role of marital status and quality on average glycemic levels among adults 50 years and older in the English Longitudinal Study of Ageing
Source: BMJ Open Diabetes Res Care. 2023 Jan 3;11(1):e003080. doi: 10.1136/bmjdrc-2022-003080 (PMC9930545; doi:10.1136/bmjdrc-2022-003080)
Supplement: Supplementary data [file bmjdrc-2022-003080supp001.pdf]

```
***MAIN ANALYSIS***

local func = "typ"

foreach x of local func {

xtreg hba1c `x'sps age, fe i(idauniq)

        est store model_1_`x'

        matrix table = r(table)

        matrix list table

        putexcel C3=(_b[`x'sps]) using "MarR1.xlsx", sheet("`x'") modify

        putexcel C4=(table[5,1]) using "MarR1.xlsx", sheet("`x'") modify

        putexcel E4=(table[6,1]) using "MarR1.xlsx", sheet("`x'") modify

xtreg hba1c `x'sps age income work bmi depress phyact smoke, fe i(idauniq)

        est store model_2_`x'

        matrix table = r(table)

        matrix list table

        putexcel H3=(_b[`x'sps]) using "MarR1.xlsx", sheet("`x'") modify

        putexcel H4=(table[5,1]) using "MarR1.xlsx", sheet("`x'") modify

        putexcel J4=(table[6,1]) using "MarR1.xlsx", sheet("`x'") modify

xtreg hba1c `x'sps typfam typfrd typchd age income work bmi depress phyact smoke, fe i(idauniq)

        est store model_3_`x'

        matrix table = r(table)

        matrix list table

        putexcel M3=(_b[`x'sps]) using "MarR1.xlsx", sheet("`x'") modify

        putexcel M4=(table[5,1]) using "MarR1.xlsx", sheet("`x'") modify

        putexcel O4=(table[6,1]) using "MarR1.xlsx", sheet("`x'") modify

xtreg hba1c `x'sps typfam typfrd typchd age income work bmi depress phyact smoke [pw=weight], fe
i(idauniq)

        est store model_4_`x'

        matrix table = r(table)

        matrix list table
```

```
		putexcel R3=(_b[`x'sps]) using "MarR1.xlsx", sheet("`x'") modify
		putexcel R4=(table[5,1]) using "MarR1.xlsx", sheet("`x'") modify
		putexcel T4=(table[6,1]) using "MarR1.xlsx", sheet("`x'") modify
		}

preserve
keep if typsps==1
local func = "sup str"
foreach x of local func {
xtreg hba1c `x'sps age, fe i(idauniq)
		est store model_1_`x'
		matrix table = r(table)
		matrix list table
		putexcel C3=(_b[`x'sps]) using "MarR1.xlsx", sheet("`x'") modify
		putexcel C4=(table[5,1]) using "MarR1.xlsx", sheet("`x'") modify
		putexcel E4=(table[6,1]) using "MarR1.xlsx", sheet("`x'") modify
xtreg hba1c `x'sps age income work bmi depress phyact smoke, fe i(idauniq)
		est store model_2_`x'
		matrix table = r(table)
		matrix list table
		putexcel H3=(_b[`x'sps]) using "MarR1.xlsx", sheet("`x'") modify
		putexcel H4=(table[5,1]) using "MarR1.xlsx", sheet("`x'") modify
		putexcel J4=(table[6,1]) using "MarR1.xlsx", sheet("`x'") modify
xtreg hba1c `x'sps `x'fam `x'frd `x'chd age income work bmi depress phyact smoke, fe i(idauniq)
		est store model_3_`x'
		matrix table = r(table)
		matrix list table
		putexcel M3=(_b[`x'sps]) using "MarR1.xlsx", sheet("`x'") modify
		putexcel M4=(table[5,1]) using "MarR1.xlsx", sheet("`x'") modify
```

```
        putexcel O4=(table[6,1]) using "MarR1.xlsx", sheet("`x'") modify
xtreg hba1c `x'sps `x'fam `x'frd `x'chd age income work bmi depress phyact smoke [pw=weight], fe i(idauniq)
        est store model_4_`x'
        matrix table = r(table)
        matrix list table
        putexcel R3=(_b[`x'sps]) using "MarR1.xlsx", sheet("`x'") modify
        putexcel R4=(table[5,1]) using "MarR1.xlsx", sheet("`x'") modify
        putexcel T4=(table[6,1]) using "MarR1.xlsx", sheet("`x'") modify
        }
restore
```
